# Applying Seaweed Compounds in Cosmetics, Cosmeceuticals and Nutricosmetics

**DOI:** 10.3390/md19100552

**Published:** 2021-09-29

**Authors:** Lucía López-Hortas, Noelia Flórez-Fernández, Maria D. Torres, Tania Ferreira-Anta, María P. Casas, Elena M. Balboa, Elena Falqué, Herminia Domínguez

**Affiliations:** 1 Centro de Investigaciones Biomédicas (CINBIO), Departamento de Enxeñería Química, Universidade de Vigo (Campus Ourense), Edificio Politécnico, As Lagoas, 32004 Ourense, Spain; luclopez@uvigo.es (L.L.-H.); noelia.florez@uvigo.es (N.F.-F.); matorres@uvigo.es (M.D.T.); ta.ferreiraan@gmail.com (T.F.-A.); mariapc@uvigo.es (M.P.C.); elenamba@uvigo.es (E.M.B.); 2 Departamento de Química Analítica, Universidade de Vigo (Campus Ourense), Edificio Politécnico, As Lagoas, 32004 Ourense, Spain; efalque@uvigo.es

**Keywords:** marine macroalgae, ingredients, additives, bioactives, nutricosmetics

## Abstract

The interest in seaweeds for cosmetic, cosmeceutics, and nutricosmetics is increasing based on the demand for natural ingredients. Seaweeds offer advantages in relation to their renewable character, wide distribution, and the richness and versatility of their valuable bioactive compounds, which can be used as ingredients, as additives, and as active agents in the formulation of skin care products. Bioactive compounds, such as polyphenols, polysaccharides, proteins, peptides, amino acids, lipids, vitamins, and minerals, are responsible for the biological properties associated with seaweeds. Seaweed fractions can also offer technical features, such as thickening, gelling, emulsifying, texturizing, or moistening to develop cohesive matrices. Furthermore, the possibility of valorizing industrial waste streams and algal blooms makes them an attractive, low cost, raw and renewable material. This review presents an updated summary of the activities of different seaweed compounds and fractions based on scientific and patent literature.

## 1. Introduction

Consumer preferences towards green and eco-friendly products have increased in the last few years [1,2]. This trend is also found in cosmetics, which represent a competitive and rapidly changing global market demanding natural, safe, and efficient ingredients for the development of novel skin care products [3,4,5]. Other relatively new products are cosmeceuticals and nutricosmetics. The term cosmeceutical is used to define active and safe products developed and tested by the cosmetics industry to provide benefits to skin appearance and are effective for preventing and treating different dermatologic conditions [6] by offering a variety of functions [6,7,8]. A number of active ingredients, including vitamins, phytochemicals, enzymes, antioxidants, and essential oils, are also considered [9], and can be used for the formulation of creams, lotions, ointments, or masks. The use of cosmeceuticals has drastically risen in the last few years [10], in a market that also incorporates other less-traditional population segments, such as men and children [11,12,13]. Both cosmetics and cosmeceuticals have to be safe, efficient, and have good sensorial quality features [6,14]; nutricosmetics also require optimal characteristics. For the optimal development of these products, cooperation in areas such as biotechnology, chemistry, food technology, pharmaceutical technology, and toxicology is needed [15].

Marine resources represent a widely available and promising source of unique and active compounds with the potential to produce cosmetics, cosmeceuticals, and nutricosmetics. Among them, seaweeds represent a sustainable and renewable resource, gaining increasing attention for these applications [16,17]. Furthermore, valorization of waste seaweeds, such as beach-casts, which are disposed of in landfills without commercial value, could represent an attractive low-cost source for cosmeceutical industries [18]. Similarly, the valorization of invasive species could contribute to the creation of natural and eco-friendly ingredients for the cosmetic industry [19] while also contributing to the restoration of affected environments. Regardless the origin and type of seaweed, the development of environmentally-friendly sustainable extraction methods, allowing a low extraction time, minimum usage of solvents, higher extraction yields, and quality, are increasingly demanded [5,19,20,21,22].

The use of seaweed-derived ingredients in cosmetic products has increased in recent years as a result of the many scientific studies that have proved the potential skincare properties of seaweed bioactives [23,24]. Among those biologically active molecules, carotenoids, fatty acids, polysaccharides, phlorotannins, vitamins, sterols, tocopherol, phycobilins, and phycocyanins have attracted attention [9,25,26,27,28,29]. Such rich compositions have converted seaweed into potential ingredients in classical cosmetics, such as solid soaps, to replace sodium lauryl sulfate/sodium laureth sulfate [22], but many algal extracts have also been used in nutritional supplements, cosmetics, and alternative medicines recommended for skin-related diseases [30]. In this latter case, they are added as the active ingredient, because they can provide a variety of activities, including photoprotective, moisturizing, antioxidant, anti-melanogenic, anti-allergic, anti-inflammatory, anti-acne, anti-wrinkling, antimicrobial, antiaging, whitening, etc. [16,31,32,33]. Furthermore, they exhibit low cytotoxicity and low allergen contents [34].

Excellent comprehensive reviews on the subject have recently been published, including on the chemical diversity and unique properties of algal bioactive molecules or extracts for cosmetic uses [5,16,28,33,35,36,37] and the progress made in the application of bioactives from marine organisms as cosmeceuticals [27,38,39]. Most of these have emphasized the importance and scientific evidence of algae-derived compounds and their benefits, as well as current application in the cosmetic industry and their challenges and limitations in the development of cosmeceuticals [3,23,24,29,34,40]. Others have reported on particular components, such as carbohydrates [41,42], or the specific beneficial actions on hyperpigmentation, photoaging, and acne [2,23,24], as well as on perspectives for the development of greener extraction methods [35], particularly those using safe solvents [3].

The present review tries to update the advances in this field, presenting an initial section summarizing the activities of algal components of particular relevance for cosmetic and cosmeceutical formulations and then by trying to offer the multiple and faceted benefits and functions that these seaweed components can provide to products where they can be incorporated as ingredients and additives, conferring other textural, functional, and sensorial properties. The potential applications are presented based on information in the scientific literature, but also using patents claiming the use of algae and algal components.

## 2. Seaweed Components and Bioactivity

### 2.1. Polysaccharides

Seaweeds contain an important carbohydrate fraction forming part of their cell walls and these polysaccharides are specific to each type of algae: in brown alginate, laminaran and fucoidan; in green ulvan and in red agar, carrageenan is the most important. Polysaccharides are receiving increasing attention for their biofunctional and physicochemical characteristics [43]. Sulfated polysaccharides are highly interesting due to their health benefits and biological activities [32,44,45,46,47,48,49,50]. A key aspect of these polysaccharides is the close relationship between the activity and their composition and structure, particularly, their molecular weight. Therefore, depolymerization is usually proposed to enhance the activity [51], but other structural modifications can also be performed. Simple hydrophobization reactions, such as esterification, acylation, alkylation, amidation, or cross-linking reactions on native hydroxyl-, amine, or carboxylic acid functions can also enhance bioactivity [52]. Examples of these activities are summarized in Table 1.

Alginates composed of chains of d-mannuronic acid and l-guluronic acid are found in brown seaweeds. These compounds show other properties in relation to cosmetics and well-being products, particularly anti-allergic properties [32,71], an action that is also observed in formulations of hydrogels with alginate [72], and can also prevent obesity [73,74]. Laminarin does not form viscous solutions and has prebiotic [75,76], antioxidant [77,78,79], and anti-photoaging and regenerative [69] properties. Based on the wound healing [80] properties of laminarin sulfate, novel hydrogel systems have been developed [81,82,83]. In addition, promising outcomes have been exhibited in several biomedical applications, such as tissue engineering, cancer therapies, antioxidant, and anti-inflammatory properties [84]. Degradation by irradiation can enhance the radical scavenging capacity and inhibitory activity against melanin synthesis in melanoma cells [2,59].

Fucoidans are heteropolysaccharides with fucose and other monosaccharides, such as xylose, galactose, mannose, and glucuronic acid, as well as other components, mainly sulfate, uronic acids, and acetyl groups. Fucoidans offer promising potential as cosmetic ingredient [34,85,86] since they are non-toxic, biodegradable, and biocompatible [87,88], and they present a wide variety of biological properties [23,24,49,61,89,90,91]; they also reduce antioxidant and antiradical properties [23,24,34,67,92], depending on the molecular weight and sulfate content [93,94]. Fucoidans have shown confirmed benefits for preventing and treating skin photoaging and have in vitro inhibition of UVB-induced collagenase and gelatinase activities, ex vivo inhibition on elastase activity in human skin [2,64,66,95], inhibition of wrinkle-related enzymes and enhanced collagen synthesis in human dermal fibroblasts [67], and anti-inflammatory action in relation to extracellular matrix degradation by matrix metalloproteinases [27,32,42,65,96].

Sulfated polysaccharides from green algae (rhamnans, arabinogalactans, galactans and mannans) present variable compositions and structures and some properties are highly influenced by the molecular weight in terms of antiradical and chelating properties [97,98,99,100]. Ulvans are highly complex and variable sulfated polysaccharides from ulvales, composed mainly of rhamnose, xylose, glucose, glucuronic acid, iduronic acid, and sulfate [34,89,101,102]. Ulvans exhibit a variety of activities, including gelling [101,103], anti-aging [51], anti-hyperlipidemic and antiherpetic properties [71,104].

Agar is mainly composed of β-d-galactopyranose and 3,6-anhydro-α-l-galactopyranose units with variable amounts of sulfate, pyruvate, and uronate substituents. Agar has pharmaceutical and industrial cosmetic applications, including its use as a thickener and as an ingredient for tablets or capsules to carry and release drugs [105,106]. Carrageenans are generally recognized as safe (GRAS) and are approved for food applications, and are high-molecular-weight sulfated linear polysaccharides with a backbone of alternating 3- α-d-galactopyranose and 4-β-d-galactopyranose with anhydrogalactose residues [54,55,107,108]. Porphyran is a complex sulfated galactan found in *Porphyra* sp. with interesting therapeutic properties. These polysaccharides have uses as gelling agent, nutritional supplement, with antioxidants [109,110,111,112], and are antiallergic [32,113], show tyrosinase inhibitory activity [62], protection against ultraviolet B radiation [59], anti-inflammatory and antitumoral activity, and can promote the growth of beneficial bacteria in intestinal microbiota [76,113] without toxicity in mice models [114,115]. Agaro-oligosaccharides (AOS) and carrageenan-oligosaccharides (COS) present enhanced biological properties compared to native ones, in relation to prebiotic, antitumoral, and antioxidant actions, related to their chemical structure, molecular weight, degree of polymerization, and the flexibility of the glycosidic linkages [116].

### 2.2. Proteins, Peptides and Aminoacids

Some seaweeds are a rich source of proteins, their cultivation offers a higher protein yield per unit area (2.5–7.5 tons/Ha/year) compared to terrestrial crops, but their successful extraction is largely influenced by the presence of polysaccharides, such as alginates in brown seaweed or carrageenans in red seaweed [117]. Seasonal variations and habitat affect the proteins, peptides, and amino acids contents in seaweed; generally, red algae (Rhodophyceae) have higher contents (up to 47%) than green (Chlorophyceae) (between 9–26%), whereas brown (*Phaeophyceae*) have a lower concentration (3–15%) [73,118,119,120]. The proteins in the three groups of macroalgae contain all essential amino acids, and non-essential amino acids are also present [25,121,122,123]. Protein and bioactive peptides from seaweed show many health benefits and have high antioxidant properties, mainly in molecules with low molecular weights, which are also considered safer than synthetic molecules and have reduced side effects [3,124,125,126,127].

Bioactive peptides usually contain 3–20 amino acid residues and both their amino acid composition and the sequence influences their activities, such as antioxidant and antimicrobial activities, among others of pharmacological interest [128,129,130,131]. Carnosine, glutathione, and taurine are peptides with antioxidant and chelating properties [132]. Due to the lack of a carboxyl group, taurine is not a “true” amino acid but has a number of health-promoting properties, being accumulated in the thalli of several red algae, such as *Ahnfeltia plicata*, *Euthora cristata*, and *Ceramium virgatum* [133]. The peptide, PPY1, is composed of five amino acids and is obtained by enzymatic hydrolysis from *Pyropia yezoensis*, and it shows anti-inflammatory effects through the suppression of inflammatory cytokines [134]. The peptides, PYP1-5 and Porphyra 334, extracted from *Porphyra yezoensis* f. coreana Ueda showed an increase in elastin and collagen production and a decrease in the expression of matrix metalloproteinases (MMP) [135]. Ultrasound-assisted enzymatic hydrolysis has also been proposed for the successful extraction of iodinated amino acids from *Palmaria palmata* and *Porphyra umbilicalis* (red seaweeds) [136].

Mycosporine-like amino acids (MAAs) are secondary metabolites synthesized for protection against solar radiation [28,137,138]. They consist of cyclohexenone or cyclohexenimine chromophore with various amino acids, mainly glycine or iminoalcohol groups, as substituents and show antioxidant and photoprotective properties [3,137,139,140,141,142,143,144]. Among the most abundant compounds, mainly in Rhodophyceae shinorine, porphyra-334, palythine, asterina-330, mycosporine-glycine, palythinol, and palythene have been described [145,146], and their contents are dependent on the geographic, seasonal and bathymetric conditions, increasing during summer and decreasing with water depth [147]. A multifunctional cosmetic liposome formulation containing UV filters, vitamins (A, C, and E), Ginkgo biloba extract (rich on quercetin), and Phorphyra umbilicalis extract (rich in proteins, vitamins, minerals and mainly in MAA’s porphyra-334 and shinorine) was efficient against signs of aging [148] by increasing hydration and reducing wrinkles and skin roughness. Leandro et al. [149] incorporated an extract of *Asparagopsis armata* (ASPAR’AGE™) containing MAA molecules in lotions with anti-aging properties, a hydrolyzed extract Aosaine^®^ (three-quarters of aosaine consists of amino acids that are very similar those responsible for skin elasticity) extracted from *Ulva lactuca,* which present anti-aging, anti-wrinkle and stimulation of collagen properties. An extract (rich in minerals, trace elements and amino acids) from *Gelidium corneum* improves skin softness and restores elasticity. Therefore, MAAs have different properties, such as serving as natural sunscreens, possess antioxidants, anti-inflammatory, and anti-aging, and are stimulators of skin renewal, activators of cells proliferation, etc., making them a promising and safe option for pharmaceutical and cosmetic industries [150] (Table 2).

Due to the toxic effect of several synthetic dyes and the high consumer demand for natural colors in food, pharmaceuticals, cosmetics, and textile industries there has been increasing interest in the use of phycobiliproteins in the food (C-phycocyanin) and cosmetic fields (C-phycocyanin and R-phycoerythrin). Phycobiliproteins are a class of water-soluble compounds composed of proteins that are covalently bound to linear tetrapyrroles, known as phycobilins, with fluorescent properties and high molecular weights and can be used for reddish colorings [28,118,160,161,162,163]. B-phycoerythrin resists changes in pH, possesses antioxidant properties [164], and can be used as a pink or purple dye in cosmetics [165]. Phycobilins can be red (phycoerythrins) or blue (phycocyanins and allophycocyanins) and phycocyanin is usually the major pigment microalgae (*Spirulina* spp.), whereas the characteristic red color of Rhodophyta phyla is due to both the phycoerythrin and phycocyanin pigments. Phycobiliproteins (concretely, R-phycoerythrin, phycocyanin, and allophycocyanin) extracted from *Gracilaria gracilis* presented high antioxidant and radical scavenging activities, primarily when harvested in winter [157], and the extraction can yield up to 46.5% of R-phycoerythtin using an aqueous solution of ionic liquids (cholinium chloride) to remove it from fresh algal biomass [166]. Saluri et al. [167] studied *Furcellaria lumbricalis* and *Coccotylus truncatus* and found an exponential correlation between R-phycoerythrin and allophycocyanin concentrations and collection depth. The contents of phycoerythrin and phycocyanin were slightly higher and lower, respectively, for dried commercial *Porphyra* spp. extracts in comparison to *Spirulina* spp. [168].

### 2.3. Phenolics and Terpenoids

Phenolic compounds are secondary plant metabolites with a basic structure with one or more aromatic rings, presenting one or more attached -OH groups. They are synthesized as part of the defense mechanisms in plants. Phlorotannins are secondary metabolites of phloroglucinol (1,3,5-trihydroxybenzene), are structurally less complex than terrestrial tannins, and are found in polymerized structures with ether, phenyl or 1,4-dibenzodioxin linkages [169,170].

Phlorotanins are increasingly considered for cosmeceutical applications, based on their antioxidative [171,172,173,174,175], anti-allergic [27,176,177,178,179], anti-inflammatory [27,180,181], tyrosinase inhibitory [182,183,184,185,186], and antidiabetic [175] activities. Skin protection against UV irradiation was confirmed in mouse skin models [187,188]. Phlorotannins also attenuated the expression of MMP-1 (an interstitial collagenase mainly responsible for the degradation of dermal collagen in human skin aging process) [27,28,189]. Dioxinodehydroeckol from *Ecklonia cava* proved to be an effective repair agent for skin damage against UVB [190]. On the other hand, fucofuroeckol-A derived from the brown seaweed *Ecklonia stolonifera Okamura*, exhibited protective activity against UVB radiation [191]; other studies also exhibited similar results for eckol and dieckol [192,193]. A correlation between the antioxidant activity and the hyaluronidase inhibitory capacity with higher molecular weight phlorotannins was observed [172], a behavior that was also observed in other works [194,195,196]. Some properties of brown algal phlorotannins are summarized in Table 3.

Meroterpenoids exhibited antioxidant properties and can prevent skin photoaging without the risk of cytotoxicity [205]. Other meroterpenoid derivatives have also shown interesting properties in relation to protection from cell damage caused by UVA irradiation [206] and photodamage attenuation on irradiated cells [207]. In addition, the hypopigmenting effect of meroterpenoids has been associated with brown algae [208].

### 2.4. Lipids

Seaweed present a low lipidic content (usually under 5%), but they are highly unsaturated and the ω3:ω-6 fatty acids ratio is highly favorable [73,209,210]. Among the most abundant fatty acids are γ-linolenic acid, arachidonic acid, eicosapentanoic acid, and docosahexanoic acid, but other lipid types, such as sterols and phospholipids, are also found [211,212]. The main sterols found are fucosterol, isofucosterol, and clionasterol [213,214]. Several biological properties have been associated with lipids [211,215]. Polyunsaturated fatty acids (PUFA) can benefit skin barrier protection and other biological functions can be enhanced; nutricosmetics could contribute an anti-obesity effect [211,216,217] and the regulation of inflammatory responses [25,218]. Being structural components of cell membranes, sterols regulate membrane fluidity and permeability and other properties, such as antioxidant, antiproliferative, and anti-photodamage, and anti-inflammatory effects have been reported for fucosterol [28,188,219,220,221]. An effect against the malarial parasite *Plasmodium falciparum* has been exhibited [222]. The viability of human keratinocytes irradiated with UVB was not affected when cells were incubated with fucosterol, and a marked decrease in UV-irradiated MMPs and increased type-I procollagen production were observed [28,206]; other authors obtained results consistent with these observations [49,61]. Phospholipids, mainly made up of fatty acids containing a phosphate group and a simple organic molecule, have been reported to help with carotenoid absorption [223]; in other work, authors showed a reduction of body weight and fat mass in mice drinking water with lipid capsules prepared using phospholipids [224]. In addition, seaweed essential oil has been evaluated, and Rexliene and Sridhar reported the antimicrobial and anti-dandruff properties of red seaweed *Portieria hornemannii* essential oil [225]. Subsequently, an antibacterial film was created with a carrageenan biopolymer blended with extracted seaweed essential oil, showing adequate bio-physical, mechanical, and anti-microbial properties. Table 4 summarizes the biological activities associated with lipids.

### 2.5. Vitamins

Vitamins obtained from diet and through topical application are essential for many functions of human skin. Supplementation is considered for protection against dehydration and premature aging of the skin, cosmetic prevention of damage by sun exposure, regulation of the secretory activity of the sebaceous glands, and the preservation of the anatomical integrity of adnexial structures [232]. Vitamins are popular ingredients in many cosmeceuticals and skin care products. Vitamins A, C, E, K and vitamin complex B are the most important and clinically validated for skin photoaging prevention and treatment [233] and the common vitamins in algae are vitamins A, B, C, and E [3,16,25,234].

Vitamin A or the retinol form shows antioxidant and antiwrinkle capacity [37,235,236] and is topically used in cosmetics to reduce facial hyperpigmentation and fine wrinkles [237]. The concentration of vitamin complex B (B_1_, B_2_, B_3_ or niacine, B_6_, B_9_ or folic acid, B_12_) is generally higher in green and red seaweeds [3,238]. Vitamin B_3_ active forms added to skin care products include: niacinamide, nicotinic acid nicotinate esters. Niacinamide is an antioxidant, reduces hyperpigmentation (also due to blue light-induced), and improves aspects of the epidermis by reducing the trans-epidermal water loss [7,239]. Red algae and other species are good sources of vitamin B_12_ for vegetarians; this vitamin shows anti-aging properties and is essential for hair and nail growth and health [25,240,241,242].

Vitamin C is used in the cosmeceutical industry as it is an l-ascorbic acid of which the biologically active form is most known [236]. The red algae *Ceramium rubrum* and *Porphyra leucosticta* show high vitamin C or ascorbate content. This vitamin, topically applied, has antioxidant, detoxifying, antiviral, anti-inflammatory, antimicrobial, and anti-stress effects, and can be used for enhancing tissue cell growth, repairing blood vessels, teeth, and bones [7,243]. Many studies reported skin improvements in fine lines and reduction of pigmentation and inflammation if present in an appropriate concentration in a cosmetic formula [7,244]. Several works confer tyrosinase inhibition to vitamin C due to it interacting with copper ions, which reduces melanogenesis [236].

Vitamin E (α, ã, ä tocopherol), the most abundant fat-soluble vitamin of non-saponifiable lipids in many algae, can be extracted from different green, red, or/and brown seaweeds [245], and is effective against UV damage, photoaging, and skin cancer when is in a high concentration and in a non-esterified form [209,246]. Cosmetic formulations usually include vitamin C since it regenerates oxidized vitamin E [7]. Vitamin K, found in high concentrations in some seaweeds, has well-known blood clotting properties (wound, bruises, marks, and scar healing) [247,248,249].

### 2.6. Minerals

Seaweeds have a high mineral content, about 8–40% [250,251,252], and this wide range is dependent on seaweed phylum and species, seaweed oceanic residence time, geographical locations, wave exposure, and seasonal and annual environmental factors [234]. Seaweeds possess most of the mineral elements from the sea, and their content depends on the pH, temperature, and the concentration of the minerals in seawater. Seaweeds have been described as an ideal safe natural source of minerals. Inorganic ions play important roles in different functions of the skin, whereas others can be considered dangerous as a consequence of dermal exposure [253]. Table 5 shows the average mineral content in different type of seaweeds.

Seaweeds contain a variety of mineral elements, macro-elements, and trace elements, which are an excellent mineral source for cosmeceutical benefits for humans. Several minerals (e.g., Ca, Fe, Mg, P, Na, Zn, Cu, and Se) are recognized as necessary for health and well-being. All seaweeds contain high amounts of both macro minerals (Ca, Mg, Na, K, and P) and trace elements (Fe, Zn, I, Cu, Se, and Mn) [234,250,255]. High potassium contents were reported in red macroalgal *Gracilaria* species and the brown macroalgal species *Laminaria digitata*; nevertheless, seaweeds have low Na/K ratios (<1.5) [250]. *Caulerpa veravelensis*, *Ulva lactuca,* and *Sargassum polycystum* contain higher amounts of calcium. Seaweeds have been described as a good source of iodine, which is present in several chemical forms, and brown algae contains greater amounts, up to over 1% wet weight; its accumulation in seaweed tissues could be 30,000 times its concentration in sea water [254,255]. According to Peñalver and coauthors, seaweeds are a primary source of iodine, allowing to achieve daily iodine requirements [234], as it is an essential element in order to maintain thyroid function and health [234,251].

Polefka et al. summarized the scientific evidence available on the benefits and risks of topical application of mineral salts [256]. Seaweeds are, in general, a better source of minerals than sea salts, because the proportion of minerals are closer to those in human skin and body’s plasm and the penetration of nutrients is better [39]. Due to this high affinity to human skin, mineral sea salts used in cosmetics are rapidly absorbed, and refresh and replenish or hydrate the skin [39,257]. Several skin care and cosmetic products contain various nutrients and minerals from seaweed, seawater, or sea mud, especially for their therapeutic properties fir psoriasis and other skin-related disorders, and for their beneficial effects on skin (they help to retain water for a longer time, restores skin pH, help in blood circulation, acne repair, and prevention, and have anti-aging effects) [39,257,258]. Alves et al. reported that high concentrated forms of marine minerals and trace elements provide a protective effect against UV radiation [259].

### 2.7. Pigments

Regarding the concentration of pigments, seaweeds are classified into three groups: green (chlorophylls a, b and c), brown (carotenoids), and red (phycobilins as phycoerythrin). In addition, free radical scavenging, inhibiting melanogenesis, and photoprotection are some of the properties of these compounds that make them suitable for skin care [260]. Carotenoids are isoprenoid molecules produced by photosynthetic plants, fungi, and algae. These lipophilic compounds can be chemically classified as carotenes, such as α-carotene, β-carotene, and lycopene, and xanthophylls. Carotenoids are used as colors in foods and as natural color enhancers, in the food, pharmaceutical, and cosmetic industries. Some act as provitamin A, and recently they have attracted considerable interest due to their antioxidant and anti-inflammatory properties [261]. β-Carotene helps to counteract free radicals involved in various diseases and premature aging [28]. In this context, the extracts obtained from three brown seaweeds were assessed to study antioxidant capacities, where fucoxanthin, violaxanthin, â-carotene, cyanidin-3-O-glucoside, and other carotenoid and chlorophyll derivatives were also characterized. The results suggest that these compounds are responsible for antioxidant properties [262].

Fucoxanthin is the main carotenoid in brown algae, this xanthophyll can counteract oxidative stress caused by UV radiation [171] and suppresses tyrosinase activity in UVB-irradiated guinea pig and melanogenesis in UVB-irradiated mice [27]; anti-melanogenic, anti-aging and antioxidant activities were also associated with this compound [40]. Fucoxanthin enhanced the fat burning rate of fat cells in adipose tissue and might be used to treat obesity and reduce the risk of certain disorders, such as type 2 diabetes [26,28,261,263,264]. Some reported actions are summarized in Table 6.

## 3. Technological Functions

**Figure 1:**
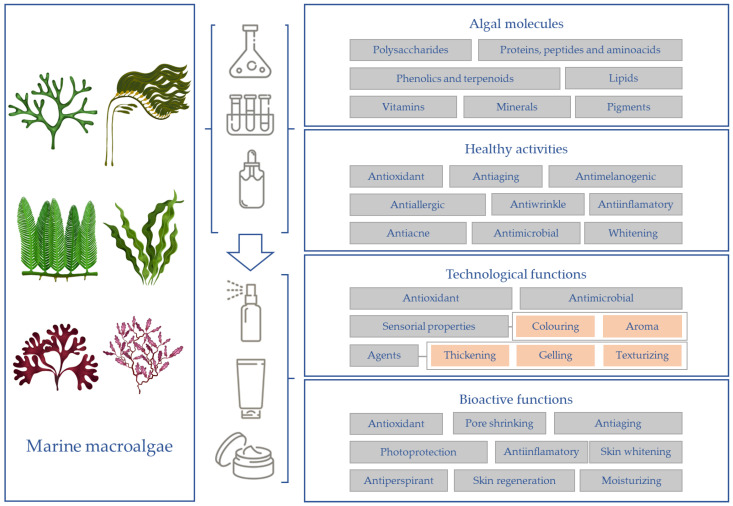
Cosmeceutical potential of algae components.

According to their functions, cosmetic ingredients are classified as (i) additives; (ii) stabilizing or excipient agents; and (iii) bioactive compounds, with real cosmetic functions [28,35]. Algal components can be used as technical ingredients to improve texture, color or stability of cosmetics, but also as bioactive agents, since they can confer a variety of biological desirable actions, which are applicable in the manufacturing of cosmeceuticals and skin care products [38,273]. Macroalgal components can be included in cosmetics as thickening or gelling agents, antioxidant, and colorants, or as active ingredients in hydrating, antiaging, skin-whitening, and pigmentation reduction products. These dual potentialities are summarized in Figure 1.

The incorporation of seaweed components was successful in different physical forms, and are commercially available in soaps, shampoos, sprays, hydrogels, or creams [274,275]. Their efficiency and stability can be enhanced with suitable carrier systems or vesicles, such as liposomes, nano/microparticles, emulsions, hydrogels, etc., designed to carry active agents in commercial products to achieve promoted effects [276,277,278].

### 3.1. Antimicrobial Agents

The antimicrobial properties of seaweed components are well known, in particular for food and pharmaceutical industries [279,280,281]. Extracts from macroalga have shown antibacterial and antifungal activities, the most active components being terpenoids and phlorotannins [27,281], which can avoid the side effects and allergic reactions associated with synthetic drugs [282]. Extracts from brown and green seaweeds proved effective against acne vulgaris [201,283], brown algal extracts against common skin pathogenic bacteria, such as methicillin-*resistant Staphylococcus aureus*, *Staphylococcus aureus* and *Staphylococcus epidermidis* [284,285,286], green algal extracts showed activity against oral bacteria [78,206], and red algae are active against *Staphylococcus* and *Candida* sp. [28,287,288,289,290]. In order to have products with a wider spectrum of protection, mixtures could be a valid approach. Widowati et al. formulated a moisturizer cream with adequate color and odor, using an antibacterial extract obtained from mixtures of *Sargassum duplicatum*, *Sargassum echinocarpum,* and *S. polycystum* extracts, which inhibited the development of bacteria for a longer period of time [291]. All seaweeds contained steroids, quinones, flavonoids, and alkaloids, and saponins were only found in *S. duplicatum*.

### 3.2. Antioxidants

Since many cosmetic and cosmeceutical formulations contain a lipidic component, they are highly susceptible to lipid peroxidation. The addition of antioxidants is needed to protect from oxidative deterioration, which also maintains the sensorial properties of the cosmetic products, in the context of appearance and odor. The contradictory data on the safety of synthetic chemical antioxidants have incentivized the search and use of natural compounds with antioxidant properties. Seaweeds represent an abundant and widespread source of compounds with confirmed antiradical and reducing properties [173,284,285,286,292]. Furthermore, they showed the potential to protect and/or retard oxidation of cosmetic products [20] and have a wide range of biological properties. The most efficient algal compounds are phlorotannin-derived fractions, but peptides and polysaccharide fractions also display reducing properties and antiradical capacity [97,293]; the phenolic compounds found in red seaweed can also scavenge free radicals and also show other properties, such as the inhibition of tyrosinase [294].

### 3.3. Sensorial Properties

The incorporation of different seaweed ingredients has to be evaluated in relation to organoleptic, spreadability, and hedonic tests [295,296]. Seaweed can provide different compounds with coloring compounds as an alternative to synthetic, mineral, and plant dyes, and show lower allergenic properties. Among the major compounds with this property are phycobilins and carotenoids, which cover a wide range of blue, yellow, orange, and red colors [29], as well as other biologically interesting properties [264].

Aroma is a key feature in cosmetics and cosmeceuticals, and the potential of seaweeds to produce terpenoids, carotenoids, fatty acid derivatives, and sulfur compounds is well known [29,297].

### 3.4. Texturizing

Thickening, gelling, and texturizing agents are used to control viscoelasticity and to form a cohesive internal structure in cosmetic products. Alginate, has been traditionally used in the cosmetic industry as a stabilizer for emulsions and suspensions due to its high stability, and for its thickening and gelling properties [56,57,171,298]. Later, authors indicated that it could be used as a hydrogel for the encapsulation of bioactives, drug delivery systems, and tissue engineering. The use of extreme pH values is not recommended and the concentration of polyvalent metal ions must be controlled in cosmetic formulations using alginates by means of adding sequestrants to avoid altering the viscosity and alginic acid precipitation [28,299]. Agar, a polysaccharide from red algae, can be applied to control both the viscosity and emollience of cosmetic products. Dita et al. confirmed that agar from *Gracilaria* sp. has a gelling agent capable of having a thickening effect on certain products, such as liquid bath soap, as a cocamide DEA (diethanolamine) substitute [300]. Carrageenans are commonly used in cosmetics as stabilizing, thickening, and gelling agents due to their excellent properties, such as gel-forming ability and chemical stability [27,30,58,301]. The rheological behavior of carrageenan and hybrid carrageenans is temperature sensitive and also depends on the structure, sulfate content, or molecular weight [28,302,303]. Carrageenans can be degraded by carrageenases to produce a number of even-numbered carrageenan oligosaccharides, which exhibit different attractive functions, such as anti-inflammation, anti-tumor, anticoagulation, or antithrombosis effects [304].

Bagal-Kestwal et al. summarized the use of carrageenans (κ, λ, and ι with sodium) as binder and emulsion stabilizers, preventing constituent separation in toothpastes in a recent comprehensive review [274]. In a previous work, these carrageenans were proposed as bodying, emulsion stabilizer, thickeners, dispersion media for shampoos, body lotion, and other cosmetic creams, and as an ingredient binder for personal lubricants [305]. Some comprehensive works [278,306] discussed the most recent breakthroughs in the field of skin care and rejuvenation using cosmeceutical facial masks developed using biopolymer-based hydrogels, which are commonly used for sensitive skins with cooling and soothing effects. Tiwari and co-workers explained the potential of biopolymers in the development of topical matrices (cream, ointment, and gel) employed as dosage forms for burn treatments [298]. Later, authors detailed the potential of carrageenans for drugs delivery or alginates for wound dressings due to their hemostatic potential. Wasupalli et al. pointed out the ability of carrageenans to form unique thermoreversible gels that are very useful to encapsulate active compounds in the cosmetic field [302]. Graham et al. corroborated the potential of thermoresponsive polymers, including agarose or carrageenan, to be used in cosmetics [307]. Hu et al. proposed a simple method to prepare hydrophilic−hydrophobic core−shell microparticles using seaweed polymers (alginates, κ-carrageenan, or agarose) with great prospective applications in the protection of unstable compounds and delivery and controlled release of drugs or bioactives in cosmetics [301].

## 4. Bioactive Functions

### 4.1. Moisturization

Moisturizer agents help to maintain skin appearance and elasticity, improving its barrier role against harmful environmental factors [28]. Polysaccharides in cosmetics are efficient at maintaining hydration, and algal extracts that are rich in polysaccharides would be an alternative to hydroxy acids [38] and are also promising for their various properties that are beneficial to skin, including antioxidant, anti-melanogenic, and skin anti-aging properties [23,24,41]. Water:propylene glycol (1:1) extracts of *Laminaria japonica* showed skin moisturizing properties in in vivo tests with human skin [28,308]. Wang et al. reported that polysaccharides from this seaweed absorbed and retained more moisture than polysaccharides from both the red algae *Chondrus cripus*, which provides hydrating, moisturizing, and therapeutic effects, and from the green algae *Codium tomentosum*, which can regulate water distribution in skin [28]. Agar is used as a moisturizer for skin and hair [46]. Mineral-rich seaweed extracts may be found in skin moisturizing agents, facial cleansing products, masks, make-up removers, bath additives, and in products to prevent cellulites. Fatty acids, either in the diet or topically applied, are efficient at preventing trans-epidermal water loss [37].

### 4.2. Skin Whitening

Skin whitening, particularly demanded in Asia, is also desired to achieve fair and flawless skin. Tyrosinase catalyzes two distinct significant reactions in melanin synthesis: the hydroxylation of l-tyrosine to 3,4-dihydroxy-l-phenylalanine, which is oxidized to dopaquinone, and further converted to melanin. Sun exposure increases the synthesis of both tyrosinase and melanosomes. Different seaweed components can be active tyrosinase inhibitors and are commonly proposed for skin whitening [27,192,193,309]; and brown algal extracts are as effective as kojic acid [284,285,286,310,311]. Similarly, Park et al. reported that *P. yezoensis* extracts could be proposed as a safe and effective agent to enhance skin whitening and prevention or alleviation of skin wrinkle formation. The extracts exhibited a significant decrease in tyrosinase activity, but was less marked than arbutin. However, arbutin could have secondary undesirable effects, whereas these aqueous seaweed extracts promoted collagen production and, in a study with 23 volunteers, they also enhanced skin brightness [312].

Due to the variety of activities, different fractions of seaweeds have been combined to achieve complementary actions, i.e., between phenolics and polysaccharides [313]. In addition, seaweed mixtures can be explored for their dermo-cosmetic potential [195,291,314,315], i.e., a cream mask from a mixture of seaweeds showing antibacterial, cell proliferation, moisture retention, and tyrosinase inhibitory activities, and also high spread and adhesive abilities, being a nonirritant and safe [314]. In addition, combination with other marine ingredients, such as nanomelanin from *Halomonas venusta*, isolated from a marine sponge *Callyspongia* sp., incorporated in a cream fortified with concentrates of seaweed *Gelidium spinosum* showed antioxidant, antimicrobial, and wound healing activity in addition to improved texture [316].

### 4.3. UV Protection, Antioxidant and Antiaging

Skin aging, causing thinning, dryness, laxity, fragility, enlarged pores, fine lines, and wrinkles, is a complex process of intrinsic and extrinsic aging. Intrinsic aging refers to the natural degradation of the skin, whereas extrinsic aging results from reactive oxygen species (ROS) generated during exposure to UV radiation [28]. Although the human body possesses an endogenous antioxidant system able to block reactive oxygen species, under conditions of oxidative stress, these defenses can be insufficient and may lead to free radical cell damage to proteins, lipids, and DNA. ROS accumulation may be responsible for photoaging complications, such as cutaneous inflammation, erythema, premature aging, melanoma, and skin cancer [317]. UVB-induced decreased cell viability could be restored by eckstolonol treatment through the enzymatic activities of catalase and superoxide dismutase [200]. Ultraviolet B irradiation induces the production of matrix metalloproteinases, and is structurally and functionally related to zinc endopeptidases, capable of digesting extracellular matrix components, such as collagens, proteoglycans, fibronectin, and laminin [64,90]. Sun-damaged skin shows significantly elevated levels of active gelatinases than intrinsically aged skin, since prolonged exposure to UV radiation causes the enzymatic breakage of collagen and elastin fibers, which are responsible for maintaining the elasticity and integrity of skin [6]. Bioactive compounds derived from marine sources [29] and from algae, especially phlorotannins, have potential anti-photoaging agents, preventing UV-induced oxidative stress, and also inhibit the expressions of MMPs in human dermal fibroblasts [27,176,318]. Riani et al. reported antioxidant and anti-collagenase activity of a *Sargassum plagyophyllum* extract as active pharmaceutical ingredient for anti-wrinkle cosmetics [319]. The potential of fucoxanthin was also confirmed, and its incorporation was compatible with other components in homogeneous water creams [295].

Since these compounds are preferentially extracted in organic solvents, different examples of macerated extracts with potential photoprotective action can be found [4,204,320,321]. Since other compounds, such as mycosporine-like amino acids, sulfated polysaccharides, carotenoids, and polyphenols, exhibit photoprotective action though a wide range of biological activities, including ultraviolet absorbing, antioxidant, matrix-metalloproteinase inhibitors and anti-aging activities, crude extracts with complex composition can be promising [322]. Gager et al. reported that the phlorotannin-enriched fractions, extracted by maceration and further purified by a liquid–liquid extraction showed antioxidant and photoprotective activities comparable to those of commercial molecules and the anti-aging activity of the obtained fraction was higher than that of epigallocatechin gallate [204]. The efficiency of mixtures of components has been described. Hameury et al. [323] confirmed that an association of ingredients from marine origin revealed activity on the epidermis and the dermis, by regulation of proteins involved in gene expression, cell survival and metabolism, inflammatory processes, dermal extracellular matrix synthesis, melanogenesis and keratinocyte proliferation, migration, and differentiation, thus helping to prevent the visible signs of skin aging.

## 5. Patents

Seaweeds and their components have been claimed as functional, sensorial, and biological agents in the formulation of cosmetics, cosmeceuticals, and nutricosmetics. Some representative examples on their utilization in the formulation of products with different claimed actions are summarized in Table 7.

Seaweeds can be used either fresh or fermented [349,363,399], and are usually incorporated as extracts, but also a pure single compounds, such as P-334 and DP-334 from *Porphyra dentata* [378], can be found. Both single species and seaweed mixtures have been found [359,386,387,400]. In addition, seaweed extracts can be combined with extracts from terrestrial plants, medicinal herbs, mushroom, microalgae, and fish [401,402,403,404,405,406], as well as with conventional ingredients [332,350,351,407,408,409,410] or even gold [372]. These mixtures of species and combination with other raw materials during manufacturing of cosmetics can be adopted to generate synergistic effects [335,359,375,411].

A variety of formulations has been found, including liposomes [335] and nano-liposome emulsions for improving the stability of extracts and its compatibility in the cosmetic system, reaching a deep layer of skin and minimizing sensitization responses by direct contact with epidemic cells [334]. Not only have creams been the object of patents, but other specific products, such as masks [412], disposable glove-shaped hand films [357], or mist compositions with fine particles that are widely dispersed when sprayed [413].

Seaweed fractions can confer functional properties or technical properties, such as thickening [346] and emulsifying properties of the polysaccharides, alginate, agar, and carrageenan [324], which also can impart water retention ability, conferring a smooth or moist humectant feeling without imparting stickiness [325,329]. Particularly, in hair cosmetics, they can provide a glossy and elastic feeling for hair and a moist feeling for the scalp [333,350]. Sensorial properties, such as suppressing the stickiness or stiffness of hair, facilitating hairdressing, the extensibility and spread on hair and producing a good feeling in its use, which are desirable for these types of products [330]. Seaweed components can also replace different additives, such as antimicrobials [342] or conventional ultraviolet ray blockers [382].

Seaweed components are interesting in the formulation of different hygienic products, including deodorants, shampoos [330,349,414,415], and cleaning supplies [328,416], especially water washing-free cleaning agents without surfactants. Other proposed formulations of cosmetics were aimed at skin condition [417] and moisturizing [403,410] improvements. Cosmeceuticals containing seaweeds are non-irritants [376], and can perform different functions, such as improving psoriasis and preventing skin problems, especially atopic dermatitis [393], hyperpigmentation [375,401,402,408], acne [410], wrinkles [406], and hair loss [351,401,402]. Many products formulated claims of a plurality of skin care effects, i.e., moisturizing, repair, and anti-aging [338,339,418] or melanin-formation inhibitory action, alleviation of skin stains and freckles, amelioration of roughened and dry skins, and conferring skins gloss and tension [387].

A number of nutricosmetics have been designed to be used in common foods and beverages to improve skin appearance and firmness [386,387], but also claim to improve immunity, strengthening the body’s constitution and improving skin antioxidant capacity [368], losing weight, and beautifying skin [419].

Other patents have claimed pollution-free, safe, sanitary, ecological, environmentally friendly and energy-saving preparation processes [338,339,374,377,419] and also purification and deodorization stages [380,419,420,421,422,423].

## 6. Conclusions and Future Trends

Cosmetics, cosmeceuticals, and nutricosmetics are daily-use products that are gaining increasing commercial importance for improving the appearance of skin and for treating various dermatologic conditions. Seaweeds are a source of valuable components for the formulation of products due to the variety of functional, sensorial and biological properties they can confer. A diverse group of biologically active compounds, including vitamins, minerals, amino acids, carbohydrates, and lipids, can be extracted from seaweeds to develop conventional and novel cosmeceutical products. The possibility of offering a vast array of activities makes seaweeds a highly attractive renewable and versatile resource, and the importance of extraction and purification processes should also be considered. Other important aspects requiring study are in relation to greener extraction of bioactives, their chemical and biological characterization, as well as stabilization and delivery into novel products. As with other ingredients and applications, quality control and standardization are required for the commercial use of seaweed bioactives.

## Figures and Tables

**Table 1:** Some activities and properties of seaweeds polysaccharides of interest in cosmeceutical formulations.

Component	Properties/Activities	Seaweed	References
Agar	Thickener; antioxidant	*Pterocladia*, *Pterocladiella*, *Gelidium amansii, Gracilaria*	[28,46,53,54,55]
Alginate	High stability, thickening agent, gelling agent	Brown seaweeds	[34,56,57]
Carrageenans	Antioxidant, antitumor, antiaging, thickeners properties, radiation protection	Red seaweeds, *Porphyra haitanensis*, *Gracilaria chouae*, *Gracilaria blodgettii*	[16,49,58,59,60,61,62]
Fucoidans	Photoaging inhibition; minimized elastase activity; antioxidant, anti-inflammatory collagenase and elastase inhibition, skin-whitening	Fucoidan (Sigma), *Ascophyllum nodosum*, *Chnoospora minima*, *Ecklonia maxima*, *Hizikia fusiforme, Saccharina japonica*, *Sargassum hemiphyllum*, *Sargassum horneri*, *Sargassum polycystum*, *Sargassum vachellianum*	[2,23,24,43,44,46,48,63,64,65,66,67]
Laminaran	Reconstructed dermis; skin cell anti-inflammation; antioxidant	*Saccharina longicruris*, Laminarin (Sigma)	[68,69]
Polysaccharides	Hydration	*Saccharina japonica*, *Chondrus crispus*, *Codium tomentosum*	[28]
Ulvan	Antiaging, antiherpetic	*Ulva pertusa*, *Ulva* sp.	[51,70]

**Table 2:** Some activities and properties of seaweeds protein, peptides, and amino acids of interest in the cosmeceutical formulations.

Extract/Compound	Activity	Seaweed	Reference
Eleven mycosporine-like amino acids	UV-protective effect, antioxidant	*Agarophyton chilense*, *Pyropia plicata* and *Champia novae-zelandiae*	[147]
Mycosporine-like amino acids extract (with porphyra-334 and shinorine in a ratio of 2:1)	Anti-aging	*Phorphyra umbilicalis*	[151]
Mycosporine-like amino acids extract (mainly palythine and asterina-330)	Antioxidant, UV-protective effect, anti-aging	*Curdieara covitzae, Iridaea cordata*	[152]
Mycosporine-like amino acids extract (mainly porphyra-334, shinorine, palythine and asterina-330)	Antioxidant; UV-protective effect	*Gracilaria vermiculophylla*	[153]
Mycosporine-like amino acids extract (mainly palythine, asterina-330, shinorine, palythinol, porphyra-334 and usujirene)	Antioxidant, antiproliferative	*Chondrus crispus*, *Mastocarpus stellatus*, *Palmaria palmata*	[154]
Mycosporine-like amino acids extract (mainly deoxygadusol, palythene and usujirene)	Antioxidant	*Rhodymenia pseudopalmata*	[155]
Aqueous extract from freshwater macroalga (mainly polysaccharides and amino acids)	Skin moisturizing effect	*Rhizoclonium hieroglyphicum*	[156]
Peptide PPY1	Anti-inflammatory	*Pyropia yezoensis*	[134]
Peptides PYP1-5 and porphyra 334	Increase production of elastin and collagen	*Porphyra yezoensis* f. coreana Ueda	[135]
Methanol extract rich in proteins, vitamins, minerals, porphyra-334 and shinorine	Hydration, skin protective, anti-wrinkle, anti-roughness	*Phorphyra umbilicalis*	[148]
Phycobiliproteins (R-phycoerythrin allophycocyanin and phycocyanin)	Antioxidant	*Gracilaria gracilis*	[157]
Hydrolyzed extract	Antitumor	*Porphyra haitanesis*	[158]
Algae extract	Decrease of progerin production, anti-elastase, anti-collagenase	*Alaria esculenta*	[159]

**Table 3:** Examples of recent studies confirming the phlorotannin activities of interest for cosmeceutical products.

Compound	Activity	Seaweed	References
Dioxinodehydroeckol	Preventive activity against UVB-induced apoptosis	*Ecklonia cava*	[190]
Dieckol	Adipogenesis inhibitory effect	*Ecklonia cava*	[197]
Eckol	Anti-inflammatory, anti-tyrosinase	*Eisenia bicyclis, Ecklonia stolonifera*	[192,193,198]
Eckol, 6,6′-bieckol, 8,8′-bieckol, dieckol, and phlorofucofuroeckol-A	Antiallergic	*Ecklonia cava*, *E. stolonifera*	[179]
Fucofuroeckol-A	Protective against UVB	*Ecklonia stolonifera Okamura*	[191]
Fuhalol	Antioxidant	*Cystoseira compressa*	[175]
Fucophloroethol (isomer)	Antioxidant	*Fucus vesiculosus*	[199]
Eckstolonol	Antioxidant enzymatic activities of catalase and superoxide dismutase	*Ecklonia cava*	[200]
Octaphlorethol A	Antioxidative effects	*Ishige foliacea*	[201]
Phlorofucofuroeckol A	Hepatoprotective effect against oxidative stress	*Eisenia bicyclis*	[93]
	Tyrosinase inhibitory activity	*Ecklonia stolonifera*	[182]
2-phloroeckol and 2-*O*-(2,4,6-trihydroxyphenyl)-6,60-bieckol	Tyrosinase inhibitory activity	*Ecklonia cava*	[185]
Phlorofucofuroeckol B	Antiallergic	*Eisenia arborea*	[202]
Phlorotannins	Antioxidant, anticoagulant, antiinflammatory, antibacterial, antiviral, antitumor; antidiabetic, photoprotective	Brown algae, *Ascophyllum nodosum*, *Fucus serratus*, *Himanthalia elongata*, *Halidrys siliquosa*	[45,172,175,198,203,204]

**Table 4:** Activities of seaweed lipids of interest in the formulation of cosmeceuticals.

Compound	Activity	Seaweed	References
E-10-oxooctadec-8-enoic acid, E-9-oxooctadec-10- enoic acid	Anti-inflammatory	*Gracilaria verrucosa*	[226]
Essential oil (tetradeconoic acid, hexadecanoic acid, (9Z, 12Z)-9,12-octadecadienoic acid, (9Z)-hexadec-9-enoic acid)	Antibacterial activity against *Staphylococcus aureus* and *Bacillus cereus* Antioxidant: radical scavenging (DPPH, superoxide, ABTS)	*Laminaria japonica*	[227]
Fucosterol	Antioxidant: increased antioxidative enzymes (superoxide dismutase, catalase, glutathione peroxidase)	*Pelvetia siliquosa*	[219,228]
Fucosterol	Anti-photodamage: decreased UVB-induced MMPs and increased procollagen Anti-inflammatory	*Hizikia fusiformis*	[60,188]
Phytosterol	Antitumoral	Commercial (Sigma)	[229]
Lipidic profile	Antioxidant, enzyme inhibition	*Ulva rigida*, *Gracilaria* sp., *Fucus vesiculosus*, *Saccharina latissima*	[211]
Unsaturated fatty acids	Antioxidant	Brown algae	[230]
Fatty acid profiling	Bioindicator of chemical stress	*Pterocladia capillacea*, *Sargassum hornschuchii*, *Ulva lactuca*	[231]

**Table 5:** Minimum and maximum values (g/100 g or mg/kg dry weight) for macro and micro elements found in edible European macroalgae [234,254].

Element (Concentration)	Brown Algae	Green Algae	Red Algae
Ca (%)	0.89–1.32	0.21–1.87	0.39–45.0
Mg (%)	0.22–1.2	0.12–2.8	0.20–167
P (%)	0.15–0.98	0.21–500	0.10–1.40
K (%)	3.8–11.5	1.1–8.1	0.33–10.2
Na (%)	1.3–7.1	0.52–8.9	1.1–4.3
S (%)	1.33–1.5	0.23–8.5	1.5–4.0
Cu (ppm)	1.1–11.0	1.6–12.1	<0.4–35.0
I (ppm)	0.20–500	20–1000	0.24–1200
Fe (ppm)	15.8–270	17.7–2890	16–1820
Mn (ppm)	<1–52.7	<2–347	<1–748
Zn (ppm)	2.5–52.3	1.98–84	7.2–714.4

**Table 6:** Biological activities of algal pigments of interest in cosmeceuticals.

Extract/Compound	Activity	Seaweed	References
97% fucoxanthin extract	Antioxidant (DPPH scavenging capacity, reducing power)	*Himanthalia elongata*	[265]
Fucoxanthin	Antioxidant, anti-melanogenesis	*Brown seaweeds*	[266,267]
	Antiobesity	*Undaria pinnatifida*	[263]
	Skin protective (antiphotodamage, anti-pigmentary, antiphotoaging, anti-wrinkling	*Sargassum siliquastrum*	[268]
	Anti-inflammatory	*Myagropsis myagroides*	[269]
	Tyrosinase activity	*Laminaria japonica*	[266]
	Photoprotective	*Undaria pinnatifida*	[270]
	Antioxidant	*Sargassum fusiforme,*	[271]
Lutein	Whitening; visual disorders and cognition diseases	*Rhodophyta* spp.	[28,272]

**Table 7:** Examples of patents claiming the use of seaweed and seaweed components in cosmetic, cosmeceutical, and nutricosmetics formulations to confer different properties.

Activity	Applicant Company	References
Functional and sensorial		
Emulsifying, water retention, gelling	Asahi Denka Kogyo Kk; Health Care Ltd.; Ichimaru Pharcos Inc; Iwasekenjiro Shoten Kk; Lg Household & Amp Lvxinyan Guangdong Bio Tech Co. Ltd.	[324,325,326,327]
Film forming	Kowa Techno Search Kk	[328]
Improved water solubility and imparting excellent feeling of use	Artnature Co. Ltd.; Kanebo Ltd.; Koosee Kk; Kyoei Kagaku Kogyo Kk; Natura Cosmeticos Sa; Toyo Shinyaku Co. Ltd.;	[329,330,331,332,333]
Stabilization system	Yantai New Era Health Industry Daily Chemical Co. Ltd.	[334]
Biological		
Anti-aging and antistress	Givenchy Parfums; Hanbul Cosmetics Co. Ltd.; Hainan Hairun Biolog Technology Co. Ltd. Shengfeng Yantai Agricultural Tech Co. Ltd.	[335,336,337,338,339]
Anti-inflammatory	Explzn Inc; Yantai Yucheng Enterprise Man Consulting Co. Ltd.	[340,341]
Antimicrobial	Nippon Enu Yuu Esu Kk	[342]
Antioxidant	Gelyma; Jeollanamdo	[343,344]
Antiperspirant, desodorant	Japan Natural Lab Co. Ltd.	[345]
Anti-wrinkle	Mamachi Co. Ltd.	[346]
Bood circulation	Kowa Techno Search Kk	[328]
Hair and scalp care and treatment, hair growth	Clean Sea Co. Ltd.; Henkel Ag & Co Kgaa; Kose Corp; Nantong Snakebite Therapy Res Inst; Pinebio Co. Ltd.; Sako Kk; Shirako Co. Ltd.; Lion Corp	[347,348,349,350,351,352,353,354]
Moisturizing	Amazonebio Co. Ltd.; Clarins; Jingmen Nuoweiying New Material Tech Co. Ltd.; Kracie Home Products Ltd.; Qingdao Better Biolog Science & Technology Co. Ltd.	[308,355,356,357,358]
Oil control, acne prevention and removal of acne marks	Yantai New Era Health Ind Daily Chemical Co. Ltd.; Guangzhou Yuanmeisheng Cosmetic Co. Ltd.; Shanghai Bonaquan Cosmetics Co. Ltd.; Suzhou Cosmetic Materials Co. Ltd.; Tubio; Yantai New Era Health Ind Daily Chemical Co. Ltd.	[359,360,361,362,363]
Pore shrinking, cleaning and minimizing	Foshan Aai Cosmetic Health Care Product Co. Ltd.; South China Sea Inst Oceanology; Rongding Guangdong Biotechnology Co. Ltd.; Kose Corp	[364,365,366]
Prevention and amelioration of aged and rough skin	Anhui Shuanglu Flour Co. Ltd.; Dzintars As; Explzn Inc; Guangzhou Saliai Stemcell Science & Technology Co. Ltd.; Kyoei Chemical Ind; Nox Bellcow Zs Nonwoven Chemical Ltd.; Shanghai Bonaquan Cosmetics Co. Ltd.; Wuhu Chuanshi Information Tech Co. Ltd.	[360,367,368,369,370,371,372,373]
Protecting from pollution	Codif International Sa	[374]
Safe melanin production and whitenning	Ichimaru Pharcos Inc; Shenzhen Sanda Cosmetics Co. Ltd.	[375,376]
Skin regeneration and epidermal cell repair	Beihai Yuanlong Pearl Company Ltd.; Jeonnam Bioindustry Found; Hexie Tech Co. Ltd.; Mokpo Marin Food Industry Res Center; Yantai New Era Health Industry Daily Chemical Co. Ltd.	[334,377,378,379]
Sunscreen and anti-sun tan	Lg Household & Health Care Ltd.; Mikimoto Seiyaku Kk; Miin	[380,381,382]
Weight-reduction and slimming	Kanebo Ltd.; Sekisui Plastics	[383,384]
Whitening	Ichimaru Pharcos Inc; Lion Corp; Mikimoto Seiyaku Kk; World Costec Co. Ltd.	[385,386,387,388,389]
Mixed Effects, More Than One Of The Following Actions		
Antiaging, anti-allergic, anti-inflammatory, antioxidant, anti-wrinkle; cleaning, moisturizing, repairing, sunscreen, whitenning	Amorepacific Corp; Baiyun Lianjia Fine Chemical Factory; Ecomine Co. Ltd.; Foshan Chancheng Relakongjian Biotechnology Co. Ltd.; Guangdong Danz Group Co. Ltd.; Guangzhou Baiyun Lianjia Fine Chemical Factory; Guangzhou Keneng Cosmetic Res Co. Ltd.; Guangzhou Xibao Daily Chemical Co. Ltd.; Hainan Shiboli Biotechnology Co. Ltd.; I2b Co. Ltd.; Jeollanamdo; Kaiso Shigen Kenkyusho Kk; Pola Chem Ind Inc	[344,390,391,392,393,394,395,396,397,398]

## Data Availability

Data are available in the original papers cited.
